# Lunacy Certification in Scotland

**Published:** 1907-05-18

**Authors:** 


### The Hospital

A Journal of

The Medical Sciences and Hospital Administration.

New Series. No. 7, Vol. I. [No. 1079, Vol. XLII.] SATURDAY, MAY 18, 1907.

### LUNACY CERTIFICATION IN SCOTLAND.

The position in which the medical profession in Scotland is placed in relation to the certification of lunatics is an eminently unsatisfactory one. Indeed, it involves personal risks to which no member of the community ought to be asked voluntarily to subject himself. In such circumstances it is inevitable that individual practitioners will decline to accept these risks, and as a result a certain number of lunatics who ought in the interests of the safety both of themselves and others to be under restraint, will remain free to exercise their dangerous possibilities. It is necessary to say this at the outset in order to make it quite clear that this is not merely a question which concerns the doctors. On the contrary, it is a matter of much more importance to the public than it is to the medical profession. And if the public can only be aroused to the dangers inherent in the present position, we cannot doubt that they will promptly take decided action with a view to their own protection. For it is quite certain, that so long as a medical practitioner is liable, as he is in present circumstances, to suffer the risks and expenses of an action for damages whenever a lunatic who has been certified by him is released from an asylum, so long will members of the profession be forced to protect themselves by abstaining from accepting the responsibility which the granting of a lunacy certificate involves. There is, be it understood, no question here of the practitioner's honesty and good faith, and no suggestion of conspiracy or malice. In the exercise of his best judgment, with all due care, and in accordance with the requirements of the law, a medical practitioner certifies a person to be insane. This is a step taken directly in the public interest. It requires, therefore, public protection. In the present circumstances that protection is not provided. Unless it is provided medical practitioners will be compelled to consult their own immediate interests and safety and to leave insane persons to such control as can be exercised by relatives and by the chance interference of the police.

The protection here demanded for the medical profession can readily stand upon the justness of its own claim. But it receives support from the attitude which the State adopts in these very same cases to those who follow the profession of the law. And the direct responsibility for the confinement of a lunatic, it must be remembered, is taken, not by the medical practitioners concerned in the case, but by the judicial authorities. What the medical practitioners who sign the necessary certificates do in substance is to act as witnesses. Their evidence is submitted to the judicial officer appointed for that purpose, and it is upon his order and under his authority that the alleged lunatic is removed to and confined within an asylum. Yet in the event of the lunatic recovering his sanity he cannot, on his release from the asylum, bring an action against the sheriff (the judicial officer concerned in these cases in Scotland) who has authorised his detention. The sheriff's action is privileged, and is therefore not liable to be called in question on the initiative of any private suitor. But the medical practitioner enjoys no such protection. He may be sued for damages, and even though the verdict is given in his favour he will be made to suffer much anxiety and loss of time, not to speak of the frequent impossibility of obtaining the payment of his costs as decreed against the unsuccessful suitor. The argument may be placed on still higher ground. The medical certificate of lunacy, as has just been stated, is essentially a piece of written evidence. Had this evidence been given by word of mouth and in the witness-box of the sheriff's court it, like the sheriff's judgment, would have been privileged; it could not, therefore, have been made the basis of an action against the witness. But under the present law, though the written certificate of the medical practitioner is essentially evidence, and evidence on which the sheriff may or may not act, though the sheriff himself is protected, and though oral evidence generally is privileged, the medical witness in these cases is left exposed to all the risks above described in the event of the lunatic at any time regaining his freedom. Such a position is fundamentally unjust, and until it is altered medical practitioners cannot be blamed for declining to act in cases of suspected lunacy.

That the propositions here stated are actual and not merely academic is painfully evident from the facts of a recent case. Two medical practitioners certify a patient to be insane, and, on the order of a sheriff, the patient in due course is removed to an asylum. Subsequently a question of his release is raised, and after an inquiry he is set free—again on a sheriff's order. He then sues each of the doctors for £10,000; a verdict with costs is given against him, but the costs cannot be recovered, that is, each of the doctors has to pay to his own legal advisers and representatives a sum of about £600. Still later they have to meet an attempt to raise a second action in the courts. In the meantime the released person takes the opportunity to fire three revolver shots at, and seriously to wound, one of the doctors. Now we are not in the least questioning the good faith or ability of the sheriff who ordered this man's detention, or of the sheriff who set him free. Nor do we for a moment suggest that anyone who believes himself to be injured as a result of these legal orders should have a right of action against the individuals who issued them. The sheriff in each case was engaged in the public service and was acting, to the best of his judgment, in the public interest. But exactly the same is true of the medical practitioners, and what we demand is that the protection already enjoyed by the legal officer should be equally extended to the medical officer. Let this be clearly presented to the public, and their sense of justice will endorse it as a demand which is necessary to their protection. For it is no light matter to members of the community to have a number of uncontrolled lunatics in their midst.

